# Impact of magnetic resonance imaging visibility of prostate cancer on partial gland ablation

**DOI:** 10.1002/bco2.70065

**Published:** 2025-08-06

**Authors:** Masatomo Kaneko, Lorenzo Storino Ramacciotti, Yuta Inoue, Samuel Peretsman, Jessica Cummins, Jie Cai, Pierre Halteh, Suzanne Palmer, Manju Aron, Osamu Ukimura, Inderbir S. Gill, Andre Luis Abreu

**Affiliations:** ^1^ USC Institute of Urology, Center for Image‐Guided Surgery, Focal Therapy and Artificial Intelligence for Prostate Cancer California Los Angeles USA; ^2^ Urology Specialists of the Carolinas Charlotte North Carolina USA; ^3^ Department of Radiology, Keck School of Medicine University of Southern California California Los Angeles USA; ^4^ Department of Radiology Cedars‐Sinai Medical Center California Los Angeles USA; ^5^ Department of Pathology, Keck School of Medicine University of Southern California California Los Angeles USA; ^6^ Department of Urology, Graduate School of Medical Science Kyoto Prefectural University of Medicine Kyoto Japan

**Keywords:** magnetic resonance imaging, partial gland ablation, PIRADS, prostate biopsy, prostate cancer

## Abstract

**Objectives:**

To evaluate the outcomes of partial gland ablation (PGA) according to prostate cancer (PCa) visibility on magnetic resonance imaging (MRI).

**Subjects and Methods:**

Consecutive patients with localized PCa diagnosed by MRI‐informed prostate biopsy (PBx), who underwent hemi‐gland Cryoablation (CRYO) or hemi‐gland High‐Intensity Focused Ultrasound (HIFU), were identified from a multicentric database. High‐visibility was defined as Prostate Imaging–Reporting and Data System (PIRADS) ≥ 4. The primary endpoint was treatment failure (TF), defined as Grade Group (GG) ≥ 2 on follow‐up PBx (FU‐PBx), any whole‐gland treatment, systemic therapy, metastases or PCa‐specific mortality. Kaplan–Meier and Cox regression analyses were performed. Statistically significant if p < 0.05.

**Results:**

A total of 156 patients met the inclusion criteria being 96 (62%) high‐visibility and 59 (38%) low‐visibility groups on baseline MRI. The baseline characteristics were as follows: median age 65yo, prostate‐specific antigen (PSA) 6.0 ng/ml, 22% with PIRADS 1–2, 16% with PIRADS 3, 44% with PIRADS 4 and 17% with PIRADS 5. The 3‐year free‐survival rates for high‐visible vs low‐visible were: TF 57% vs 83% (p = 0.002); biochemical failure (PSA nadir + 2 ng/ml) 81% vs 72% (p = 0.5); GG ≥ 2 on FU‐PBx 57% vs 85% (p < 0.001); and Radical Treatment 87% vs 85% (p = 0.9), respectively. After adjusting for confounders, the independent predictors for TF were PSA density, PSA reduction and high visibility (hazard ratio 4.83, 95% confidence interval 1.81–12.90).

**Conclusion:**

MRI visibility is an independent prognosticator for outcomes following focal therapy for prostate cancer. Patients with higher MRI visibility (PIRADS ≥4) are at an increased risk of treatment failure.

## 1 INTRODUCTION

Currently, the standard management options for clinically localized prostate cancer (PCa) are radical prostatectomy (RP), radiation therapy (RT) and active surveillance (AS). Although radical treatments are effective in treating PCa, they are associated with functional outcomes deterioration. Conversely, while AS retains patients' quality of life, it is associated with the risk of cancer progression. Partial gland ablation (PGA) for carefully selected patients with localized PCa has emerged as a promising approach, offering the potential benefits of effective cancer control while preserving functional outcomes.^1^, ^2^, ^3^

Identifying patients with a poor prognosis is essential for selecting the most appropriate treatment option. Quantitative radiomic analyses, such as apparent diffusion coefficient (ADC) metrics, dynamic contrast enhancement (DCE) and shape and texture features of prostate magnetic resonance imaging (MRI), may hold promise for risk stratification based on tumour aggressiveness.^4^ However, radiomic analysis faces challenges, including a lack of standardization, no consensus on optimal thresholds and limited multicentric validation. It has been reported that the visibility of PCa on MRI assessed by the Prostate Imaging Reporting & Data System (PIRADS), a standardized prostate MRI acquisition and interpretation system, may be associated with radical prostatectomy and active surveillance outcomes at phenotypic and transcriptomic levels.^5^, ^6^, ^7^, ^8^, ^9^ We hypothesized that PCa visibility assessed by PIRADS may serve as a prognosticator for patients undergoing PGA.

Herein, we evaluated oncological and functional outcomes of PGA according to PCa visibility on MRI in a multicentric cohort.

## 2 SUBJECTS AND METHODS

### 2.1 Ethics Statement

All procedures in this study were conducted in compliance with the ethical standards of the institutional (IRB# HS‐17‐00749) and/or national research committees, as well as the 1964 Declaration of Helsinki and its subsequent amendments or equivalent ethical guidelines. Informed consent was obtained from the participants.

### 2.2 Study population

Consecutive patients with localized PCa diagnosed by MRI‐informed prostate biopsy (PBx) who underwent hemi‐gland Cryoablation (CRYO) or hemi‐gland High‐Intensity Focused Ultrasound (HIFU) between January 2013 and September 2023 as an initial treatment were identified from an institutional review board‐approved multicentric database.

### 2.3 MRI Protocol

All patients underwent multipatametric MRI (mpMRI) prior to PBx. The image acquisition and interpretation were prospectively performed by radiologists and confirmed by five experienced genitourinary radiologists with 5–15 years of experience in prostate MRI reading (over 300 scans per year) following the current version of PIRADS at the time (v1 from 2013 to 2014, v2.0 for 2015 to April 2019 and v2.1 for May 2019 to September 2023).^5^, ^6^, ^7^ The image sequences consisted but were not limited to T2‐weighted, diffusion‐weighted using b100, b800 and b1400, ADC map, and DCE images. A 3 T MR with a 16‐channel phased‐array surface coil was used. Image quality was ensured during acquisition in accordance with PIRADS. MRIs obtained at the outside facilities were accepted if they met PIRADS standards, and were reviewed by the five experienced radiologists.

### 2.4 Prostate Biopsy Protocol

The patients underwent a sextant 12‐core systematic biopsy with additional two or more target biopsy cores per PIRADS 3–5 lesion using an imaging fusion software (Trinity® [Koelis, Grenoble, France] or UroNav® [Philips, Gainesville, Florida]).^2^, ^3^ Each core was independently assessed by two sub‐specialized, fellowship‐trained urologic pathologists with >20 years of experience in prostate cancer diagnosis (over 500 cases per year) according to the International Society of Urological Pathology (ISUP) guidelines.^10^ Any discrepancy between imaging and pathology was discussed in the institutional tumour board for confirmation. CSPCa was defined as ISUP Grade Group ≥2. Index PCa was defined as the PCa with the highest ISUP Grade Group and largest size.

### 2.5 Partial Gland Ablation Protocol

Hemi‐gland ablation was performed in the prostatic lobe harbouring the PCa index lesion. The localization of index PCa to a hemi‐gland was confirmed by MRI‐guided target and systematic biopsy. Low‐volume grade group (GG) 1 PCa elsewhere were left untreated. Hemi‐gland CRYO and HIFU were performed as previously described.^2^, ^3^ The PGA was performed according to the uniform treatment protocol across institutions.^11^ An argon/helium‐gas‐based system Endocare (HeathTonics Inc., Austin, USA) was used for CRYO, while Sonablate (Sonablate Corp, Charlotte, USA), Ablatherm or FocalOne (EDAP TMS, Vaulx‐en‐Velin, France) were used for HIFU.

### 2.6 Follow‐up Protocol

The follow‐up was scheduled every 3 months for the first year and every 6 months thereafter, assessing symptoms, validated questionnaires, prostate‐specific antigen (PSA) levels and digital rectal examination (DRE). MRI was performed at 6 to 12 months and then annually thereafter. Follow‐up PBx (FU‐PBx) were performed using the same technique as the baseline biopsy at 6 to 12 months as per protocol, or anytime for a cause, such as PSA rise or suspicion for recurrence by DRE or mpMRI.^2^, ^3^

### 2.7 Endpoint, Definitions and Statistical Analysis

Patients with PIRADS 1–3 were defined as Low‐visibility and PIRADS 4–5 as High‐visibility. Oncologic and functional outcomes were compared between the Low‐visibility vs the High‐visibility groups. The primary endpoint was treatment failure (TF) defined as Grade Group (GG) ≥ 2 on FU‐PBx, any whole‐gland treatment, systemic therapy, metastases or PCa‐specific mortality. Secondary endpoints were survival‐free from I) GG ≥ 2 on FU‐PBx (CSPCa recurrence); II) Biochemical Failure (BF, PSA nadir + 2 ng/ml) and III) Radical Treatment. PSA reduction was calculated as the percentage difference between pre‐PGA PSA and the post‐PGA nadir PSA. Functional outcomes were assessed pre‐ and post‐operatively by the best scores on the International Prostate Symptom Score (IPSS) and the International Index of Erectile Function 5 (IIEF5) score.^12^, ^13^ Continence was defined as no usage of the pads.^2^, ^3^ Post‐operative complications within 90 days were captured according to the Clavien‐Dindo classification.^14^ Kaplan–Meier and Cox regression analyses were performed to assess survival. In multivariable Cox regression analyses, clinical characteristics at baseline, imaging findings including prostate volume and visibility on MRI, pathologic findings at baseline MRI‐guided biopsy, D'Amico risk group and ablation modalities were evaluated as clinically relevant variables.^1^, ^2^, ^3^, ^8^, ^9^, ^10^, ^15^ Statistical significance was defined as p < 0.05. Statistics were analysed with JMP PRO 16 (SAS Institute Inc., Cary, NC, USA).

## 3 RESULTS

### 3.1 Patient Characteristics

A total of 156 males with diagnosis of localized PCa were treated with hemi‐gland PGA, being 60 (38%) in the Low‐visibility group and 96 (62%) in the High‐visibility group (Figure S1). The baseline characteristics of the whole cohort included a median age of 65yo, PSA of 6.0 ng/ml, PSA density (PSAD) of 0.17 ng/mL^2^, 22% with PIRADS 1–2, 16% with PIRADS 3, 44% with PIRADS 4 and 17% with PIRADS 5. Overall, 23% were low‐risk, 73% intermediate‐risk and 3.8% high‐risk PCa (Table 1 and Table S1). The High‐visibility group had higher PSAD (0.18 vs 0.13 ng/ml^2^; p < 0.001), a radiologically (12 vs 8 mm; p = 0.004) and pathologically (10 vs 2 mm; p = 0.003) larger index lesion, and a higher proportion of the intermediate‐risk PCa (82 vs 58%; p = 0.004) than the Low‐visibility group.

**TABLE 1:** Baseline Characteristics of Hemi‐gland Partial Gland Ablation for Prostate Cancer.

	PIRADS 1–5	PIRADS 1–3	PIRADS 4–5	P Value*
**No. of Patients, n (%)**	156 (100)	60 (38)	96 (62)	
**Age, year, median (IQR)**	65 (59–71)	64 (58–69)	65 (60–71)	0.1
**PSA, ng/ml, median (IQR)**	6.0 (4.7–7.7)	6.0 (4.5–9.1)	6.0 (4.8–8.1)	0.3
**Prostate Volume, cc, median (IQR)**	37 (29–49)	42 (33–55)	36 (25–47)	0.008
**PSA density, ng/ml** ^**2**^ **, median (IQR)**	0.17 (0.11–0.23)	0.13 (0.09–0.19)	0.18 (0.12–0.25)	<0.001
**Clinical T stage, n (%)**				0.1
**T1**	133 (85)	56 (93)	77 (80)	
**T2a**	19 (12)	4 (6.7)	15 (16)	
**T2b**	2 (1.3)	‐	2 (2.1)	
**T2c**	2 (1.3)	‐	2 (2.1)	
**MRI Findings**				
**Index lesion size, mm, median (IQR)**	12 (8–15)	8 (6.5–12.8)	12 (10–16)	0.004
**PIRADS score, n (%)**				<0.001
**1–2**	35 (22)	35 (58)	‐	
**3**	25 (16)	25 (42)	‐	
**4**	69 (44)	‐	69 (72)	
**5**	27 (17)	‐	27 (28)	
**Prostate biopsy**				
**Grade group, n (%)**				0.01
**1**	38 (24)	22 (37)	16 (17)	
**2**	96 (62)	29 (48)	67 (70)	
**3**	18 (12)	6 (10)	12 (13)	
**4**	4 (2.6)	3 (5)	1 (1.0)	
**5**	0 (0)	‐	‐	
**No. cancer positive cores, median (IQR)**	4 (2–5)	2 (1–4)	4 (3–6)	<0.001
**Maximum cancer core length, mm, median, (IQR)**	9.0 (4.9–11)	2 (1.5–4.5)	10 (7–11)	0.003
**Maximum cancer core involvement, %, median, (IQR)**	50 (25–80)	30 (12–50)	65 (40–90)	<0.001
**Risk group, n (%)**				0.004
**Low**	36 (23)	22 (37)	14 (15)	
**Intermediate**	114 (73)	35 (58)	79 (82)	
**High**	6 (3.8)	3 (5.0)	3 (3.1)	
**Ablation modality, n (%)**				0.5
**Cryoablation**	31 (20)	10 (17)	21 (22)	
**HIFU**	125 (80)	50 (83)	75 (78)	

* Comparison between patients with PIRADS 1–3 vs 4–5 on baseline MRI.

CRYO, cryoablation; HIFU, High‐Intensity Focused Ultrasound, IQR, Interquartile Range; MRI, magnetic resonance imaging; No., number; PIRADS, Prostate Imaging Reporting and Data System.

### 3.2 Oncologic Outcomes

**FIGURE 1:**
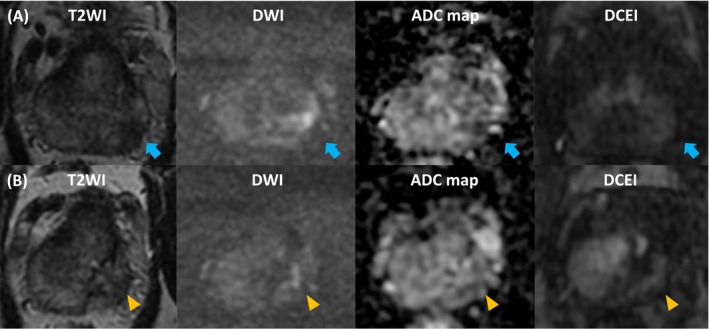
**A Representative Case with High‐Visibility.** A) A 55‐year‐old male with a PSA value of 5.3 ng/mL. Pre‐biopsy multiparametric MRI identified a 12 mm PIRADS score four lesion (blue arrowhead) at the left base peripheral zone of the prostate. Targeted biopsy toward the lesion confirmed GG 2 prostate cancer with cancer involvement of 90%. The patient underwent left hemigland HIFU. B) Follow‐up MRI at 1 year revealed a small area of focal nodular enhancement (orange triangle) in the region of the previous treatment. GG 2 prostate cancer recurrence was detected on targeted follow‐up biopsy of the lesion. The patient underwent salvage left hemigland HIFU, followed by no GG ≥ 2 recurrence over 2 years. Repeat PGA is a preferred option for unifocal GG 2–3 recurrence with limited cancer volume, long life expectancy, and the patient's desire to preserve urogenital functions.^16^ GG, Gleason grade group; HIFU, high‐intensity focused ultrasound; MRI, magnetic resonance imaging; PGA, partial gland ablation; PIRADS, Prostate Imaging Reporting & Data System; PSA, prostate‐specific antigen.

**FIGURE 2:**
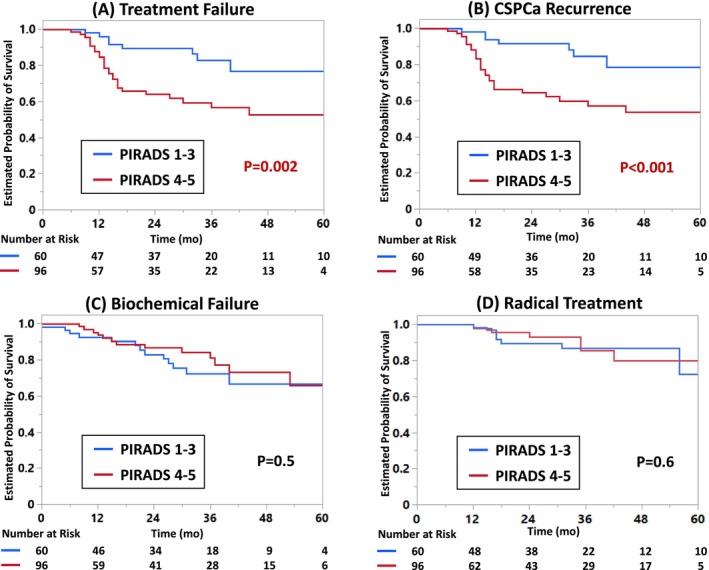
**Survivals After Partial Gland Ablation.** A) Treatment Failure Free Survival. B) Clinically Significant Prostate Cancer (Grade Group ≥2 on follow‐up biopsy) Recurrence Free Survival. C) Biochemical Failure (PSA nadir + 2 ng/ml) Free Survival. D) Radical Treatment Free Survival. The blue line and the red line represent the Low‐visibility group (PIRADS 1–3) and the High‐visibility group (PIRADS 4–5), respectively. PIRADS, Prostate Imaging Reporting & Data System; PSA, prostate‐specific antigen.

In a median follow‐up of 28 months, the oncologic outcomes were as follows: PSA reduction 78%, PSA nadir 1.4 ng/ml with a median time to nadir of 4 months, the 3‐yr free survival from treatment failure was 68%, biochemical failure 77%, CSPCa recurrence 69% and radical treatment 86% (Table 2 and Figure 1, 2).^16^ The High‐visibility group presented shorter follow‐up (23 vs 33mo; p = 0.04), a greater PSA reduction (82 vs 66%; p < 0.001), a lower PSA nadir (1.2 vs 1.7 ng/ml; p = 0.004), shorter survivals from treatment failure (p = 0.002) and CSPCa recurrence (p < 0.001). On multivariable analysis, PSAD (hazard ratio [HR] 1.03, 95% confidence interval [CI] 1.002–1.05; p = 0.01), high‐visibility on baseline MRI (HR 6.13, 95%CI 2.19–17.18; p < 0.001) and PSA reduction (HR 0.96, 95%CI 0.95–0.98; p < 0.001) were independent predictors for treatment failure (Table 3). Overall, 95 (61%) patients underwent ≥1 FU‐PBx. A sensitivity analysis was performed on patients with versus without FU‐PBx. The baseline and follow‐up parameters were similar except for follow‐up length between patients who did not undergo FU‐PBx per protocol vs those in whom FU‐PBx did not detect CSPCa (Table S2). The significance of High‐visibility to predict TF was also confirmed in a subgroup analysis of patients who underwent FU‐PBx (Table S3–S4). Furthermore, High‐visibility was the sole independent predictor for CSPCa recurrence (HR 2.51, 95%CI 1.09–5.77; p = 0.03) on multivariable analysis (Table S5).

**TABLE 2:** Oncologic and Functional Outcomes of Hemi‐gland Partial Gland Ablation for Prostate Cancer.

	PIRADS 1–5	PIRADS 1–3	PIRADS 4–5	P value*
**No. Patients, n (%)**	156 (100)	60 (38)	96 (62)	
**Follow‐up Length, mo, median**	28 (9–42)	33 (14–46)	23 (6–41)	0.04
**PSA Reduction, %, median (IQR)**	78 (60–88)	66 (42–93)	82 (68–90)	<0.001
**PSA Nadir, ng/ml, median (IQR)**	1.4 (0.7–2.6)	1.7 (1.0–3.6)	1.2 (0.62–2.3)	0.004
**Time to PSA Nadir, mo, median**	4 (3–7)	3.5 (3–6.3)	4 (3–7.3)	0.4
**No. Patients with ≥1 Follow‐up Prostate Biopsy, n (%)**	95 (61)	37 (62)	58 (60)	1.0
**Histology on Follow‐up Prostate Biopsy, n (%)**				
**Whole Gland**				0.01
**Benign**	42 (44)	24 (65)	18 (31)	
**GG 1**	22 (23)	8 (22)	14 (24)	
**GG 2**	25 (26)	4 (11)	21 (36)	
**GG 3**	3 (3.2)	1 (2.7)	2 (3.5)	
**GG 4**	3 (3.2)	0 (0)	3 (5.2)	
**Treated Side** ^**†**^				0.09
**Benign**	70 (74)	33 (89)	37 (65)	
**GG 1**	6 (6.4)	2 (5.4)	4 (7.0)	
**GG 2**	14 (15)	2 (5.4)	12 (21)	
**GG 3**	2 (2.1)	0 (0)	2 (3.5)	
**GG 4**	2 (2.1)	0 (0)	2 (3.5)	
**Untreated Side** ^**†**^				0.3
**Benign**	58 (62)	27 (73)	31 (54)	
**GG 1**	21 (22)	7 (19)	14 (25)	
**GG 2**	12 (13)	2 (5.4)	10 (18)	
**GG 3**	2 (2.1)	1 (2.7)	1 (1.8)	
**GG 4**	1 (1.1)	0 (0)	1 (1.8)	
**CSPCa Recurrence on Follow‐up**	37 (24)	8 (13)	29 (30)	0.02
**Salvage Management After CSPCa Recurrence**				0.02
**Repeat HIFU**	11 (30)	2 (25)	9 (31)	
**Repeat CRYO**	1 (2.7)	0 (0)	1 (3.5)	
**Radical Prostatectomy**	6 (16)	5 (63)	1 (3.5)	
**Radiation Therapy**	7 (19)	1 (13)	6 (21)	
**Whole‐Gland HIFU**	1 (2.7)	0 (0)	1 (3.5)	
**Androgen Deprivation Therapy**	1 (2.7)	0 (0)	1 (3.5)	
**Active Surveillance**	7 (19)	0 (0)	7 (24)	
**Referral to External Facility**	3 (8)	0 (0)	3 (10)	
**Reason of No Treatment for CSPCa Recurrence**				0.00
**Patient's Strong Preference**	6 (86)	‐	6 (86)	
**Active Surveillance for Small GG2** ^**‡**^	1 (14)	‐	1 (14)	
**3‐Yr Free Survival** ^§^ **, %**				
**Treatment Failure**	68%	83%	57%	0.002
**CSPCa Recurrence**	69%	85%	57%	<0.001
**Biochemical Failure**	77%	72%	81%	0.5
**Radical Treatment**	86%	85%	87%	0.6
**Pre to Post IPSS Difference, median (IQR)**	1 (−2 to +5)	0 (−2 to +5.5)	1 (−1.5 to +5)	0.8
**Pre to Post IIEF5 Difference, median (IQR)**	0 (−1 to +6)	0 (−1 to +8)	0 (−1 to +5.8)	1.0
**Continence Maintained** ^‖^ **, n (%)**	154 (99)	59 (98)	95 (99)	1.0

* Comparison between patients with PIRADS 1–3 vs 4–5 on baseline MRI.

^†^ One follow‐up prostate biopsy was performed elsewhere without detailed data of the cancer cores.

^‡^ One core with 1 mm GG2 was detected on follow‐up biopsy.

^§^ P value calculated by Log‐rank test.

‖ Continence was defined as not using a pad after partial gland ablation.

CRYO, cryoablation; CSPCa, clinically significant prostate cancer; GG, Gleason grade group; HIFU, High‐Intensity Focused Ultrasound; IIEF5, International Index of Erectile Function 5; IPSS; International Prostate Symptom Score; IQR, Interquartile Range; MRI, magnetic resonance imaging; No., number; PIRADS, Prostate Imaging Reporting and Data System; PSA, prostate‐specific antigen.

**TABLE 3:** Cox Proportional Hazard Regression for Treatment Failure After Partial Gland Ablation for Prostate Cancer.

	Univariable			Multivariable		
	HR	95%CI	p‐value	HR	95%CI	p‐value
**Age, yr**	1.01	0.97–1.05	0.7			
**PSA, ng/mL**	1.10	0.996–1.19	0.04			
**PSA density** * **, ng/mL** ^**2**^	1.03	1.004–1.05	0.009	1.03	1.002–1.05	0.01
**PSA reduction, %**	0.99	0.97–0.998	0.02	0.96	0.95–0.98	<0.001
**PSA nadir, ng/mL**	1.21	1.03–1.40	0.01			
**Prostate volume, cc**	0.99	0.96–1.01	0.3			
**PIRADS**						
**3–5 vs 1–2**	3.39	1.20–9.62	0.02			
**4–5 vs 1–3**	3.21	1.46–7.07	0.004	6.13	2.19–17.18	<0.001
**ISUP Grade Group**	1.43	0.94–2.15	0.09			
**Number of PCa‐positive core**	1.15	1.03–1.27	0.01	2.77	0.48–14.21	0.2
**Maximum cancer core length, mm**	1.28	0.88–2.34	0.3			
**Maximum cancer core involvement, %**	1.01	0.995–1.02	0.2			
**Risk group**						
**High or Intermediate vs Low**	2.04	0.85–4.91	0.1			
**High vs Intermediate or Low**	1.75	0.53–5.72	0.4			
**HIFU vs CRYO**	1.51	0.60–3.79	0.4			

* PSA density was calculated per 0.01 unit.

CI, confidence interval; CRYO, cryoablation; HIFU, high‐intensity focused ultrasound; HR, hazard ratio; ISUP, International Society of Urological Pathology; PIRADS, Prostate Imaging Reporting and Data System; PSA, prostate‐specific antigen.

### 3.3 Functional Outcomes and Complications

**FIGURE 3:**
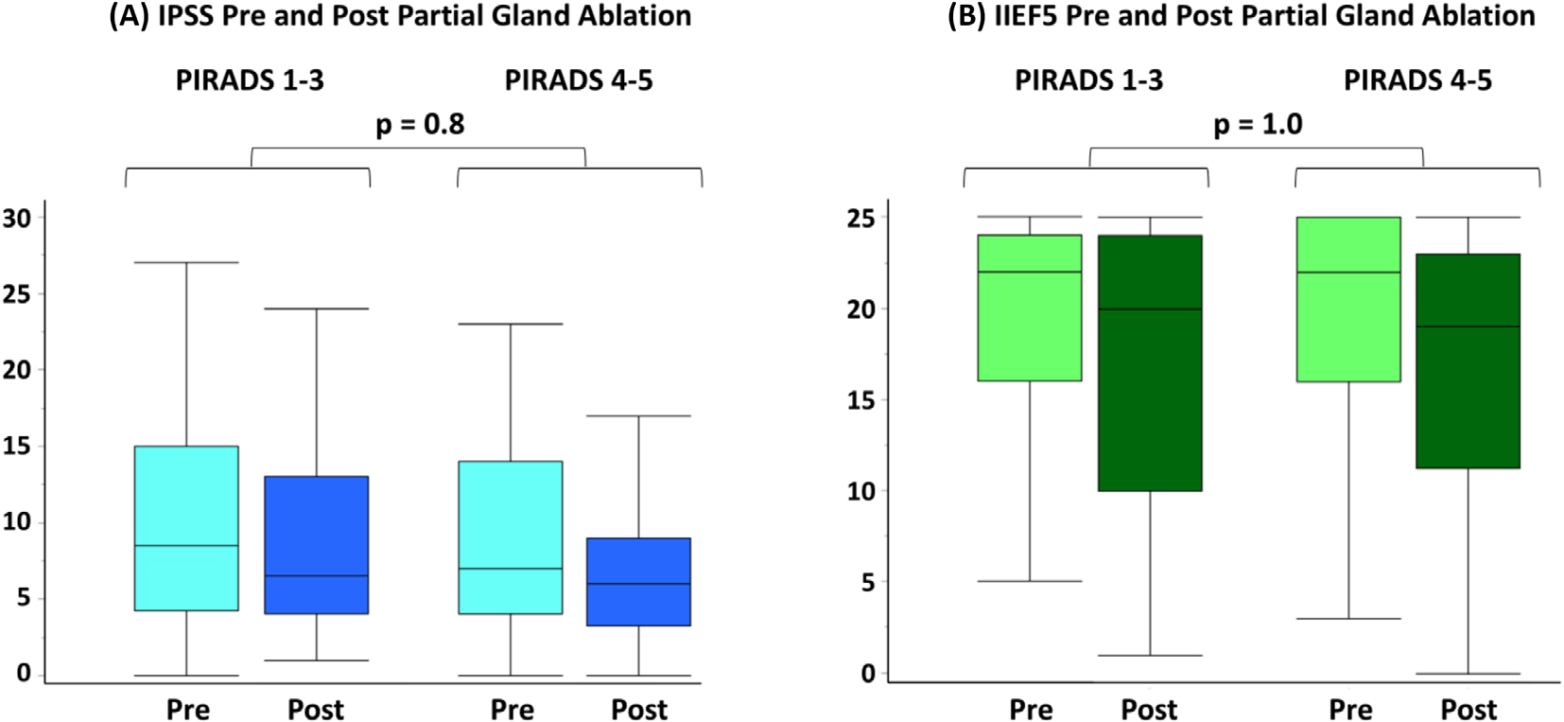
**Urogenital Functions Pre and Post Partial Gland Ablation.** A) Urinary function before and after partial gland ablation measured by the patient‐reported IPSS. The median IPSS difference from pre‐ to post‐PGA was zero for Low‐visibility and 1 for High‐visibility groups (p = 0.8). B) Sexual function before and after partial gland ablation assessed by the patient‐reported IIEF5. The median IIEF difference from pre‐ to post‐PGA was zero for both Low‐visibility and High‐visibility groups (p = 1.0). Box and whisker plots. Boxes indicate the interquartile range, and the lines inside the boxes indicate the median. IPSS, International Prostate Symptom Score; IIEF5, International Index of Erectile Function 5; PGA, partial gland ablation; PIRADS, Prostate Imaging Reporting & Data System; Post, best value within 2 years after partial gland ablation; Pre, baseline before partial gland ablation.

The median IPSS and IIEF5 difference from pre‐ to post‐PGA was 1 and zero, respectively (Figure 3). Overall, 98% maintained continence, and 9% experienced Clavien grade 1–3 complications including dysuria 5.8%, urinary tract infections 2.6% and neuropraxia 0.6% (Table 2 and Table S6). No statistical differences were observed in functional outcomes and post‐operative complications between the High‐visibility vs Low‐visibility groups.

## 4 DISCUSSION

To the best of our knowledge, this is the largest report demonstrating the impact of PCa visibility on MRI in the outcomes of PGA. All patients underwent MRI‐informed biopsies as part of their diagnostic workup prior to receiving PGA. Therefore, the reported results reflect contemporary clinical practice in the MRI era. Our study showed a significant difference in treatment failure‐free survival between the High‐visibility and Low‐visibility groups. MRI visibility may represent an independent phenotype associated with PCa prognosis. Imaging visibility of PCa might reflect an aggressive characteristic independent from those assessed by the existing ISUP GG scoring system or PSA, and, therefore, it should be considered a standalone biomarker. PCa risk stratification according to PIRADS score may improve the oncologic outcomes of current treatment options including AS, RT, RP or PGA as a valuable alternative for carefully selected patients.

In the current study, the association of PCa visibility on MRI and oncologic/functional outcomes was meticulously assessed in a multicentric and large PGA cohort. Outcomes were reported following HIFU or CRYO, which are the most commonly used modalities for PCa.^1^, ^17^ Due to a lack of agreement on the treatment margin for PGA, a pragmatic hemi‐ablation technique was employed for all included patients to maximize efficacy, reflecting a commonly used clinical approach. High‐visibility of PCa on MRI was an independent strong predictor for treatment failure post‐PGA. Furthermore, the significance of High‐visibility was robustly confirmed in a subgroup analysis restricted to patients who underwent FU‐PBx. The predictive value of High‐visibility was further confirmed for CSPCa recurrence after PGA.

Although the treatment failure‐free survival rate was lower in the High‐visibility group, the radical treatment‐free survival was comparable between the two groups. One of the key advantages of PGA is that it can be repeated if necessary. In our study, the Low‐visibility group had a higher rate of radical treatment compared to the High‐visibility group (75% vs. 28%, respectively). In contrast, the High‐visibility group showed higher rates of repeat PGA (34% vs. 25%) and AS after PGA (24% vs. 0%). A large series from the United Kingdom reported that approximately 30% of patients undergoing PGA eventually underwent a repeat PGA at 4 years.^18^ Long‐term data are needed to determine the potential for sustained avoidance of radical treatment in recurrent PCa after PGA.

Whether a low PIRADS score alone should be used to exclude patients from PGA or to recommend active surveillance remains an open and actively debated question.^19^ CSPCa may still be present even in MRI‐negative (PIRADS 1–2) cases.^20^ The significance of MRI findings in focal cryoablation for PCa has been investigated in a small (75 patients) single‐centric cohort analysis.^21^ In contrast to our findings, a significant difference was not observed between MRI‐visible vs MRI‐invisible groups regarding oncologic outcomes. However, although the difference was not statistically significant, the MRI‐invisible group showed better recurrence‐free survival than the MRI‐visible group. A potential reason for the discordant results from ours is their smaller sample size, which might be underpowered to detect the existing impact of MRI‐visibility on prognosis following PGA.

We defined PIRADS 4–5 as High visibility. Another cut‐off of PIRADS 3–5 was not an independent predictor for treatment failure after PGA. PIRADS 3 has relatively higher interreader variability than PIRADS 4–5 even when evaluated by experienced uro‐radiologists.^22^ Including PIRADS 3, a heterogeneous group, in the High‐visibility group could obscure the statistical evaluation of its prognostic value.

Recent studies have indicated a relationship between biomarkers and PIRADS score.^23^ Beksac et al. reported PIRADS score was significantly associated with Decipher® score.^23^ Additionally, PIRADS 4–5 vs 3 was an independent predictor for adverse pathology (GG > 2, pT3–4 or pN1) on RP. The PI3K‐AKT–mTOR, WNT‐β and E2F signalling pathways, known for driving cell cycle acceleration, treatment resistance and disease progression, were significantly more active in PIRADS 5 compared to PIRADS 4. These findings suggest that the MRI visibility may be driven by genomic and transcriptomic signatures, possibly correlating visibility with adverse outcomes.

Although the majority (73%) of patients in this study have intermediate‐risk PCa, this 10‐year cohort includes patients with both low‐ and high‐risk PCa, who are no longer considered ideal candidates for PGA. While a randomized controlled trial comparing AS and partial gland vascular‐targeted photodynamic therapy for low‐risk PCa demonstrated benefits of PGA, such as lower rates of cancer progression, conversion to radical therapy and detection of Gleason ≥7 on FU‐PBx,^24^ we believe that PGA should not be used as a substitute for AS in these cases. A recent meta‐analysis reported that the rates of patients with CSPCa in PGA cohorts increased substantially from 0 to 57.2% in 2013–2016 to 66.3–85.3% in 2021–2024.^25^

With the growing body of literature on PGA and a deeper understanding of the natural history of PCa, high‐risk PCa is now better managed with radical treatments. Most of the low‐ and high‐risk patients treated with PGA were from the earlier years of the study.^1^, ^13^

In the current study, the differences in risk groups or GG did not significantly impact prognosis. This may be attributed to the predominance of the intermediate‐risk group with underrepresentation of other groups. On the other hand, PSAD and PSA reduction were significantly associated with treatment failure. Reflecting pre‐treatment PCa activity, PSAD is associated with adverse pathology.^26^, ^27^ Although there is no established definition for biochemical failure following PGA, PSA kinetics including PSA reduction represents the treatment effect. Therefore, these parameters are associated with prognosis.^28^, ^29^

Overall, 61% of patients underwent at least one FU‐PBx in this cohort, which is comparable with FU‐PBx rates seen in other studies.^18^, ^25^ In clinical practice, patients without signs of recurrence may decline FU‐PBx despite strong recommendations (Table S2). In fact, a sensitivity analysis revealed that patients who did not undergo FU‐PBx had similar baseline characteristics to those who did, but they exhibited a greater reduction in PSA levels, a lower PSA nadir and fewer MRI findings suggestive of PCa recurrence. Therefore, it is expected that these patients have better oncologic outcomes. As a result, we chose treatment failure as a more practical and comprehensive endpoint.

Both the low‐visibility and High‐visibility groups demonstrated excellent functional outcomes with low complication rates, all of which were of low grade. There were minimal to no changes in urinary function, sexual function or continence when comparing pre‐ and post‐treatment assessments, with postoperative complications remaining low. These results confirm the feasibility and safety of performing PGA regardless of lesion visibility on imaging. Preserving quality of life, particularly maintaining potency and continence, is a key rationale for choosing PGA in the management of PCa.

Based on our findings, physicians should consider the PIRADS score alongside other biomarkers like PSA, ISUP GG and tumour size when formulating a treatment strategy for PCa. For patients undergoing PGA with baseline PIRADS 4–5 lesions, more aggressive approaches may be warranted. Potential strategies include PGA with wider ablation margins, combining PGA with additional treatment modalities, or opting for radical therapies, any of which may enhance oncologic outcomes.^30^, ^31^, ^32^

This study has limitations. Firstly, as a retrospective study, the results may be subject to selection bias and the influence of unknown or unmeasured confounders, despite efforts to adjust for these through multivariate analysis. Nevertheless, it is the largest multicenter study to date that successfully demonstrates a worse prognosis for highly visible PCa on MRI following PGA. Second, the results are based on the follow‐up with relatively modest length, given that most cases in the current cohort were diagnosed after the widespread use of PIRADS for PCa detection. With long‐term follow‐up data, an increase in the number of events is expected, leading to more precise estimates of hazard ratios. Lastly, given that the current study was conducted at highly experienced facilities in MRI‐informed PGA, the results may not be generalizable to facilities without experienced urogenital radiologists, focal therapists or the appropriate equipment.

## 5 CONCLUSION

Prostate cancer visibility on MRI is an independent prognosticator for oncologic outcomes following PGA. Patients with higher MRI visibility (PIRADS ≥4) are at an increased risk of treatment failure. MRI visibility should be considered an independent biomarker for PCa. Further research is warranted to refine patient selection criteria and optimize focal therapy outcomes based on MRI characteristics.

## AUTHOR CONTRIBUTIONS

*Conceptualization*: Masatomo Kaneko and Andre Luis Abreu. *Data curation*: Masatomo Kaneko, Lorenzo Storino Ramacciotti, Yuta Inoue and Jessica Cummins. *Formal analysis*: Masatomo Kaneko and Jie Cai. *Investigation*: Masatomo Kaneko, Lorenzo Storino Ramacciotti and Yuta Inoue. *Methodology*: Masatomo Kaneko and Andre Luis Abreu. *Project administration*: Andre Luis Abreu. *Resources*: Pierre Halteh, Manju Aron and Inderbir S. Gill. *Supervision*: Suzanne Palmer, Manju Aron, Osamu Ukimura, Inderbir S. Gill and Andre Luis Abreu. *Validation*: Masatomo Kaneko and Jie Cai. *Visualization*: Masatomo Kaneko. *Writing—original draft*: Masatomo Kaneko and Andre Luis Abreu. *Writing—review and editing*: Masatomo Kaneko, Lorenzo Storino Ramacciotti, Yuta Inoue, Samuel Peretsman, Jessica Cummins, Jie Cai, Suzanne Palmer, Pierre Halteh, Suzanne Palmer, Manju Aron, Osamu Ukimura, Inderbir S. Gill and Andre Luis Abreu.

## CONFLICT OF INTEREST STATEMENT

Samuel Peretsman is a paid consultant for Sonablate LLC.

Inderbir S. Gill has equity interest in OneLine Health and EditorAIpro.

Andre Abreu is proctor and speaker for Sonablate, speaker for EDAP and is proctor and has research grant with Koelis.

## Supporting information


**Figure S1.** **Patient Accrual Flow Chart.** MRI, magnetic resonance imaging.

**Table S1:** Baseline Characteristics Subdivided by Included Institutions

**Table S2:** Baseline and Follow‐up Parameters Subdivided By Follow‐up Prostate Biopsy Status

**Table S3:** Baseline Characteristics ‐Subgroup Analysis on Patients Who Underwent Follow‐up Biopsy‐

**Table S4:** Oncologic and Functional Outcomes of Hemi‐gland Partial Gland Ablation for Prostate Cancer – Subgroup Analysis on Patients Who Underwent Follow‐up Biopsy

**Table S5:** Multivariable Cox Proportional Hazard Regression for Clinically Significant Prostate Cancer Recurrence After Partial Gland Ablation for Prostate Cancer

**Table S6:** 90‐day Complications After Hemi‐gland Partial Gland Ablation for Prostate Cancer

## Data Availability

The data sets analysed during the current study are available from the corresponding author on reasonable request.
