# Supplementary material for: Impact of magnetic resonance imaging visibility of prostate cancer on partial gland ablation
Source: BJUI Compass. 2025 Aug 6;6(8):e70065. doi: 10.1002/bco2.70065 (PMC12328995; doi:10.1002/bco2.70065)
Supplement: Supplementary file 1 — Figure S1. Patient Accrual Flow Chart. MRI, magnetic resonance imaging. [file BCO2-6-e70065-s005.pdf]

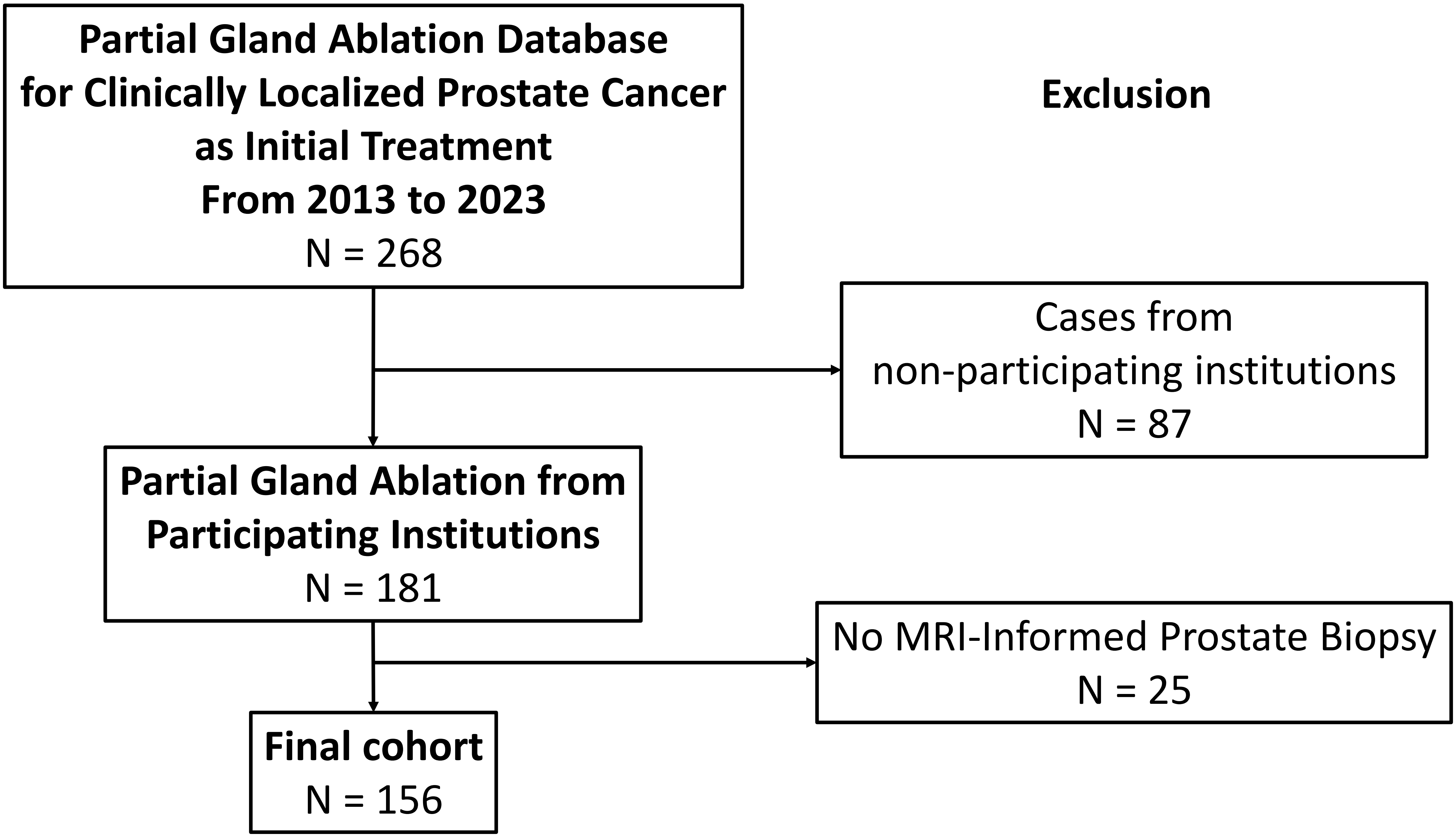
Partial Gland Ablation Database
for Clinically Localized Prostate Cancer
as Initial Treatment
From 2013 to 2023
N = 268
Exclusion
Cases from
non-participating institutions
N = 87
Partial Gland Ablation from
Participating Institutions
N = 181
No MRI-Informed Prostate Biopsy
N = 25
Final cohort
N = 156
