# Supplementary material for: Impact of magnetic resonance imaging visibility of prostate cancer on partial gland ablation
Source: BJUI Compass. 2025 Aug 6;6(8):e70065. doi: 10.1002/bco2.70065 (PMC12328995; doi:10.1002/bco2.70065)
Supplement: Supplementary file 2 — Table S1: Baseline Characteristics Subdivided by Included Institutions [file BCO2-6-e70065-s004.docx]

**Supplementary Table 1: Baseline Characteristics Subdivided by Included Institutions**

|  | Institution A | Institution B | P Value |
| --- | --- | --- | --- |
| **No. of Patients, n (%)** | 104 (67) | 52 (33) |  |
| **Age, year, median (IQR)** | 66 (60-72) | 63 (59-69) | 0.1 |
| **PSA, ng/ml, median (IQR)** | 6.2 (4.8-7.8) | 5.7 (4.6-6.9) | 0.1 |
| **Prostate Volume, cc, median (IQR)** | 39 (28-49) | 35 (30-49) | 0.8 |
| **PSA density, ng/ml^2^, median (IQR)** | 0.17 (0.11-0.24) | 0.15 (0.09-0.22) | 0.1 |
| **Clinical T stage, n (%)** |  |  | 0.004 |
| **T1** | 81 (78) | 52 (100) |  |
| **T2a** | 19 (18) | 0 (0) |  |
| **T2b** | 2 (1.9) | 0 (0) |  |
| **T2c** | 2 (1.9) | 0 (0) |  |
| **MRI findings** |  |  |  |
| **PIRADS score, n (%)** |  |  | 0.03 |
| **1-2** | 17 (16) | 18 (35) |  |
| **3** | 21 (20) | 4 (7.7) |  |
| **4** | 49 (47) | 20 (38) |  |
| **5** | 17 (16) | 10 (19) |  |
| **Prostate biopsy** |  |  |  |
| **Grade group, n (%)** |  |  | 0.2 |
| **1** | 21 (20) | 17 (33) |  |
| **2** | 67 (64) | 29 (56) |  |
| **3** | 14 (13) | 4 (7.7) |  |
| **4** | 2 (1.9) | 2 (3.9) |  |
| **5** | 0 (0) | - |  |
| **Risk group, n (%)** |  |  | 0.3 |
| **Low** | 20 (19) | 16 (31) |  |
| **Intermediate** | 80 (77) | 34 (65) |  |
| **High** | 4 (3.9) | 2 (3.9) |  |
| **Ablation modality, n (%)** |  |  | <0.001 |
| **Cryoablation** | 31 (29) | 0 (0) |  |
| **HIFU** | 73 (70) | 52 (100) |  |
| HIFU, High-Intensity Focused Ultrasound, IQR, Interquartile Range; MRI, magnetic resonance imaging; No., number; PIRADS, Prostate Imaging Reporting and Data System. | | | |
