# Supplementary material for: Impact of magnetic resonance imaging visibility of prostate cancer on partial gland ablation
Source: BJUI Compass. 2025 Aug 6;6(8):e70065. doi: 10.1002/bco2.70065 (PMC12328995; doi:10.1002/bco2.70065)
Supplement: Supplementary file 3 — Table S2: Baseline and Follow‐up Parameters Subdivided By Follow‐up Prostate Biopsy Status [file BCO2-6-e70065-s007.docx]

**Supplementary Table 2: Baseline and Follow-up Parameters Subdivided By Follow-up Prostate Biopsy Status**

|  | **Patients**  **with FU-PBx** | | **Patients**  **without FU-PBx** |  |
| --- | --- | --- | --- | --- |
|  | **CSPCa**  **on FU-PBx** | **Non-CSPCa**  **on FU-PBx** | **No FU-PBx** | **P Value*** |
| **No. of Patients, n (%)** | 37 (24) | 58 (37) | 61 (39) |  |
| **Baseline Characteristics** |  |  |  |  |
| **Age, year, median (IQR)** | 65 (58-75) | 64 (60-70) | 65 (59-71) | 0.6 |
| **PSA, ng/ml, median (IQR)** | 6.3 (5.3-8.3) | 6.1 (4.7-7.2) | 5.6 (4.7-7.1) | 0.8 |
| **Prostate Volume, cc, median (IQR)** | 36 (25-62) | 39 (30-49) | 36 (29-50) | 1.0 |
| **PSA density, ng/ml^2^, median (IQR)** | 0.18 (0.14-0.26) | 0.15 (0.1-0.21) | 0.16 (0.10-0.23) | 0.9 |
| **Clinical T stage, n (%)** |  |  |  | 0.1 |
| **T1** | 30 (81) | 51 (88) | 53 (87) |  |
| **T2a** | 6 (16) | 4 (6.9) | 8 (13) |  |
| **T2b** | 1 (2.7) | 3 (5.2) | 0 (0) |  |
| **T2c** | 0 (0) | 0 (0) | 0 (0) |  |
| **MRI Findings** |  |  |  |  |
| **Index lesion size, mm, median (IQR)** | 12 (8-15) | 11 (8-19) | 12 (10-14) | 1.0 |
| **PIRADS score, n (%)** |  |  |  | 0.3 |
| **1-2** | 5 (14) | 16 (28) | 14 (23) |  |
| **3** | 3 (8.1) | 13 (22) | 9 (15) |  |
| **4** | 20 (54) | 19 (33) | 30 (49) |  |
| **5** | 9 (24) | 10 (17) | 8 (13) |  |
| **Prostate biopsy** |  |  |  |  |
| **Grade group, n (%)** |  |  |  | 0.2 |
| **1** | 6 (16) | 19 (33) | 13 (21) |  |
| **2** | 21 (57) | 33 (57) | 42 (69) |  |
| **3** | 8 (22) | 6 (10) | 4 (6.6) |  |
| **4** | 2 (5.4) | 0 (0) | 2 (3.3) |  |
| **5** | 0 (0) | 0 (0) | 0 (0) |  |
| **No. cancer positive cores, median (IQR)** | 5 (3-7) | 3 (1-5) | 4 (2-5) | 0.5 |
| **Maximum cancer core length, mm, median, (IQR)** | 9 (5-9) | 6 (2-12) | 10 (6-11) | 0.6 |
| **Maximum cancer core involvement, %, median, (IQR)** | 50 (30-80) | 50 (25-73) | 50 (20-80) | 0.8 |
| **Risk group, n (%)** |  |  |  | 0.3 |
| **Low** | 5 (14) | 19 (33) | 12 (20) |  |
| **Intermediate** | 30 (81) | 37 (64) | 47 (77) |  |
| **High** | 2 (5.4) | 2 (3.5) | 2 (3.3) |  |
| **Ablation modality, n (%)** |  |  |  | 0.4 |
| **Cryoablation** | 8 (22) | 9 (16) | 14 (23) |  |
| **HIFU** | 29 (78) | 49 (84) | 47 (77) |  |
| **Postoperative Oncologic Outcomes** |  |  |  |  |
| **Follow-up Length, mo, median** | 36 (21-54) | 33 (20-49) | 7 (3-27) | < 0.001 |
| **PSA Reduction, %, median (IQR)** | 68 (50-83) | 77 (65-89) | 82 (64-90) | 0.5 |
| **PSA Nadir, ng/ml, median (IQR)** | 2.4 (1.2-2.9) | 1.2 (0.6-2.2) | 1.0 (0.6-2.4) | 0.7 |
| **Time to PSA Nadir, mo, median** | 3 (3-7) | 5 (3-9) | 4 (3-6) | 0.2 |
| **Suspicion on Follow-up MRI, n (%)** | 11 (32) | 7 (13) | 0 (0) | 0.2 |
| *Comparison between patients without FU-PBx vs Non-CSPCa on FU-PBx.  †The center location of the lesion with the highest PIRADS score and largest volume. In case the lesion was invisible on MRI, the location of the biopsy core with the highest Grade group.  CSPCa, clinically significant prostate cancer; FU-PBx, follow-up prostate biopsy; HIFU, High-Intensity Focused Ultrasound, IQR, Interquartile Range; MRI, magnetic resonance imaging; No., number; PIRADS, Prostate Imaging Reporting and Data System; PSA, prostate-specific antigen. | | | | |
