# Supplementary material for: Impact of magnetic resonance imaging visibility of prostate cancer on partial gland ablation
Source: BJUI Compass. 2025 Aug 6;6(8):e70065. doi: 10.1002/bco2.70065 (PMC12328995; doi:10.1002/bco2.70065)
Supplement: Supplementary file 4 — Table S3: Baseline Characteristics ‐Subgroup Analysis on Patients Who Underwent Follow‐up Biopsy‐ [file BCO2-6-e70065-s002.docx]

**Supplementary Table 3: Baseline Characteristics -Subgroup Analysis on Patients Who Underwent Follow-up Biopsy-**

|  | **PIRADS 1-5** | **PIRADS 1-3** | **PIRADS 4-5** | **P Value*** |  |
| --- | --- | --- | --- | --- | --- |
| **No. of Patients, n (%)** | 95 (100) | 37 (39) | 58 (61) |  |  |
| **Age, year, median (IQR)** | 64 (59-71) | 62 (59-68) | 65 (61-72) | 0.06 |  |
| **PSA, ng/ml, median (IQR)** | 6.2 (4.8-7.8) | 6 (4.6-6.8) | 6.2 (5.2-8.3) | 0.08 |  |
| **Prostate Volume, cc, median (IQR)** | 37 (29-48) | 42 (32-55) | 36 (25-46) | 0.04 |  |
| **PSA density, ng/ml^2^, median (IQR)** | 0.17 (0.11-0.23) | 0.13 (0.09-0.20) | 0.18 (0.14-0.3) | 0.001 |  |
| **Clinical T stage, n (%)** |  |  |  | 0.3 |  |
| **T1** | 80 (84) | 34 (92) | 46 (79) |  |  |
| **T2a** | 11 (12) | 3 (8.1) | 8 (14) |  |  |
| **T2b** | 2 (2.1) | 0 (0) | 2 (3.5) |  |  |
| **T2c** | 2 (2.1) | 0 (0) | 2 (3.5) |  |  |
| **MRI Findings** |  |  |  |  |  |
| **Index lesion size, mm, median (IQR)** | 12 (8-18) | 8 (7-12) | 13 (8-18) | 0.02 |  |
| **PIRADS score, n (%)** |  |  |  | < 0.001 |  |
| **1-2** | 21 (22) | 21 (57) | 0 (0) |  |  |
| **3** | 16 (17) | 16 (43) | 0 (0) |  |  |
| **4** | 39 (41) | 0 (0) | 39 (67) |  |  |
| **5** | 19 (20) | 0 (0) | 19 (33) |  |  |
| **Prostate biopsy** |  |  |  |  |  |
| **Grade group, n (%)** |  |  |  | 0.08 |  |
| **1** | 25 (26) | 15 (41) | 10 (17) |  |  |
| **2** | 54 (57) | 16 (43) | 38 (66) |  |  |
| **3** | 14 (15) | 5 (14) | 9 (16) |  |  |
| **4** | 2 (2.1) | 1 (2.7) | 1 (1.7) |  |  |
| **5** | 0 (0) | 0 (0) | 0 (0) |  |  |
| **No. cancer positive cores, median (IQR)** | 4 (2-5) | 2 (1-5) | 5 (3-7) | < 0.001 |  |
| **Maximum cancer core length, mm, median, (IQR)** | 8 (4-11) | 3 (2-10) | 9 (6-12) | 0.2 |  |
| **Maximum cancer core involvement, %, median, (IQR)** | 50 (30-80) | 30 (15-50) | 60 (45-83) | < 0.001 |  |
| **Risk group, n (%)** |  |  |  | 0.02 |  |
| **Low** | 24 (25) | 15 (41) | 9 (16) |  |  |
| **Intermediate** | 67 (71) | 21 (57) | 46 (79) |  |  |
| **High** | 4 (4.2) | 1 (2.7) | 3 (5.2) |  |  |
| **Ablation modality, n (%)** |  |  |  | 1.0 |  |
| **Cryoablation** | 17 (18) | 7 (19) | 10 (17) |  |  |
| **HIFU** | 78 (82) | 30 (81) | 48 (83) |  |  |
| *Comparison between patients with PIRADS 1-3 vs 4-5 on baseline MRI.  †The center location of the lesion with the highest PIRADS score and largest volume. In case the lesion was invisible on MRI, the location of the biopsy core with the highest Grade group.  HIFU, High-Intensity Focused Ultrasound; IQR, Interquartile Range; MRI, magnetic resonance imaging; No., number; PIRADS, Prostate Imaging Reporting and Data System; PSA, prostate-specific antigen. | | | | | |
