# Supplementary material for: Impact of magnetic resonance imaging visibility of prostate cancer on partial gland ablation
Source: BJUI Compass. 2025 Aug 6;6(8):e70065. doi: 10.1002/bco2.70065 (PMC12328995; doi:10.1002/bco2.70065)
Supplement: Supplementary file 5 — Table S4: Oncologic and Functional Outcomes of Hemi‐gland Partial Gland Ablation for Prostate Cancer – Subgroup Analysis on Patients Who Underwent Follow‐up Biopsy [file BCO2-6-e70065-s006.docx]

|  | PIRADS 1-5 | PIRADS 1-3 | | | PIRADS 4-5 | | P value* |
| --- | --- | --- | --- | --- | --- | --- | --- |
| **No. Patients, n (%)** | 95 (100) | 37 (39) | | | 58 (61) | |  |
| **Follow-up Length, mo, median** | 36 (20-50) | 37 (31-54) | | | 32 (19-49) | | 0.09 |
| **PSA Reduction, %, median (IQR)** | 76 (56-84) | 69 (43-82) | | | 79 (66-87) | | 0.007 |
| **PSA Nadir, ng/ml, median (IQR)** | 1.4 (0.7-2.6) | 1.7 (0.8-3.4) | | | 1.3 (0.7-2.5) | | 0.2 |
| **Time to PSA Nadir, mo, median** | 4 (3-8) | 3 (3-6.8) | | | 5 (3-10) | | 0.08 |
| **3-Yr Free Survival^†^, %** |  | |  |  | |  | |
| **Treatment Failure** | 60% | 80% | | | 48% | | 0.001 |
| **CSPCa Recurrence** | 61% | 80% | | | 48% | | 0.002 |
| **Biochemical Failure** | 72% | 66% | | | 77% | | 0.4 |
| **Radical Treatment** | 84% | 84% | | | 84% | | 0.4 |
| **Pre to Post IPSS Difference, median** | 1 (-2 to +5) | 1 (-2 to +5) | | | 1 (-1 to +5) | | 0.8 |
| **Pre to Post IIEF5 Difference, median** | 0 (-1 to +5) | 0 (-1 to +7) | | | 0 (-1 to +3) | | 0.6 |
| **Continence Maintained^‡^, n (%)** | 94 (99) | 36 (97) | | | 58 (100) | | 0.4 |
| *Comparison between patients with PIRADS 1-3 vs 4-5 on baseline MRI.  † P value calculated by Log-rank test.  ‡ Continence was defined as not using a pad after focal therapy.  CSPC, clinically significant prostate cancer; IIEF5, International Index of Erectile Function 5; IPSS, International Prostate Symptom Score; IQR, Interquartile Range; MRI, magnetic resonance imaging; No., number; PIRADS, Prostate Imaging Reporting and Data System; PSA, prostate-specific antigen. | | | | | | | |

**Supplementary Table 4: Oncologic and Functional Outcomes of Hemi-gland Partial Gland Ablation for Prostate Cancer – Subgroup Analysis on Patients Who Underwent Follow-up Biopsy -**
