# Supplementary material for: Impact of magnetic resonance imaging visibility of prostate cancer on partial gland ablation
Source: BJUI Compass. 2025 Aug 6;6(8):e70065. doi: 10.1002/bco2.70065 (PMC12328995; doi:10.1002/bco2.70065)
Supplement: Supplementary file 6 — Table S5: Multivariable Cox Proportional Hazard Regression for Clinically Significant Prostate Cancer Recurrence After Partial Gland Ablation for Prostate Cancer [file BCO2-6-e70065-s001.docx]

|  | **Univariable** | | | **Multivariable** | | |
| --- | --- | --- | --- | --- | --- | --- |
|  | **HR** | **95%CI** | **p-value** | **HR** | **95%CI** | **p-value** |
| **HIFU vs CRYO** | 1.35 | 0.56-3.26 | 0.5 |  |  |  |
| **Age, yr** | 1.01 | 0.97-1.05 | 0.6 |  |  |  |
| **PSA, ng/mL** | 1.09 | 0.99-1.19 | 0.048 |  |  |  |
| **PSA density*, ng/mL^2^** | 1.03 | 1.01-1.05 | 0.006 | 1.00 | 0.98-1.03 | 0.7 |
| **Prostate volume, cc** | 0.99 | 0.96-1.01 | 0.2 |  |  |  |
| **PIRADS** |  |  |  |  |  |  |
| **3-5 vs 1-2** | 2.87 | 1.11-7.42 | 0.03 |  |  |  |
| **4-5 vs 1-3** | 3.54 | 1.60-7.82 | 0.002 | 2.51 | 1.09-5.77 | 0.03 |
| **ISUP Grade Group** | 1.53 | 1.02-2.26 | 0.04 | 3.82 | 0.90-15.18 | 0.06 |
| **Number of PCa positive core** | 1.16 | 1.04-1.29 | 0.005 | 1.13 | 0.98-1.29 | 0.07 |
| **Maximum cancer core length, mm** | 1.12 | 0.81-1.58 | 0.5 |  |  |  |
| **Maximum cancer core involvement, %** | 1.01 | 0.998-1.02 | 0.1 |  |  |  |
| **Risk group** |  |  |  |  |  |  |
| **High or Intermediate vs Low** | 2.67 | 1.04-6.86 | 0.04 |  |  |  |
| **High vs Intermediate or Low** | 1.18 | 0.28-4.91 | 0.8 |  |  |  |
| **PSA reduction, %** | 0.99 | 0.98-1.01 | 0.2 |  |  |  |
| **PSA nadir, ng/mL** | 1.16 | 0.97-1.35 | 0.08 |  |  |  |
| *PSA density was calculated per 0.01 unit.  CI, confidence interval; CRYO, cryoablation; HIFU, high-intensity focused ultrasound; HR, hazard ratio; ISUP, International Society of Urological Pathology; PIRADS, Prostate Imaging Reporting and Data System; PSA, prostate-specific antigen. | | | | | | |

**Supplementary Table 5: Multivariable Cox Proportional Hazard Regression for Clinically Significant Prostate Cancer Recurrence After Partial Gland Ablation for Prostate Cancer**
