# Supplementary material for: Impact of magnetic resonance imaging visibility of prostate cancer on partial gland ablation
Source: BJUI Compass. 2025 Aug 6;6(8):e70065. doi: 10.1002/bco2.70065 (PMC12328995; doi:10.1002/bco2.70065)
Supplement: Supplementary file 7 — Table S6: 90‐day Complications After Hemi‐gland Partial Gland Ablation for Prostate Cancer [file BCO2-6-e70065-s003.docx]

**Supplementary Table 6: 90-day Complications After Hemi-gland Partial Gland Ablation for Prostate Cancer**

| **Clavien Grade** | **PIRADS 1-5** | **PIRADS 1-3** | **PIRADS 4-5** | **P value*** |
| --- | --- | --- | --- | --- |
| **I-III, n (%)** | 14 (9.0) | 6 (10) | 8 (8.3) | 0.8 |
| **I** | 9 (5.8) | 3 (5.0) | 6 (6.3) | 1.0 |
| **Dysuria, n (%)** | 8 (5.1) | 3 (5.0) | 5 (5.2) | 1.0 |
| **Neuropraxia, n (%)** | 1 (0.6) | 0 (0) | 1 (1.0) | 1.0 |
| **II** | 4 (2.6) | 2 (3.3) | 2 (2.1) | 0.6 |
| **Urinary tract infection, n (%)** | 4 (2.6) | 2 (3.3) | 2 (2.1) | 0.6 |
| **III** | 1 (0.6) | 1 (1.7) | 0 (0) | 0.4 |
| **Dysuria, n (%)** | 1 (0.6) | 1 (1.7) | 0 (0) | 0.4 |
| *Comparison between patients with PIRADS 1-3 vs 4-5 on baseline MRI.  PIRADS, Prostate Imaging Reporting and Data System | | | | |
